# Depression and Complicated Grief Among Parents of Pediatric Cancer Patients in Cameroon: Implications for Global Health in Low-income Countries

**DOI:** 10.1101/2025.03.28.25324833

**Published:** 2025-03-28

**Authors:** Isaac Che Ngang, Francine Kouya, Emmanuel Tetteh, Clifford Atuiri, Kaah Joel, Bienvenue Kouya, Nyinya Valery Ngalle, Vaishnavi Mamillapalle, Robert M. Chamberlain, Amr S. Soliman

**Affiliations:** 1 Department of Surgery, Washington University in St. Louis, MO, USA; 2 Mbingo Baptist Hospital, Northwest Region, Cameroon; 3 Brown School of Public Health, Washington University in St. Louis, MO, USA; 4 Department of Internal Medicine, St. Luke’s Hospital, St. Louis, MO, USA; 5 District Hospital Tiko, Southwest Region, Cameroon; 6 City University of New York (CUNU) School of Medicine, New York, NY, USA

## Abstract

**Background and Objectives:**

Bereaved parents of pediatric cancer patients frequently experience severe grief and psychological distress, but studies on the prevalence of major depressive disorder (MDD) and complicated grief (CG) among this population in Cameroon are lacking. This study aimed to determine the prevalence of MDD and CG among bereaved parents of deceased pediatric cancer patients treated at Mbingo Baptist Hospital Cameroon, and to identify predictors of these mental health outcomes.

**Methods:**

This cross-sectional study included parents of deceased pediatric cancer patients treated at Mbingo Baptist Hospital between January 2015 and January 2022. Multivariate logistic regression identified predictors of MDD and CG as adjusted odds ratios (AOR) with 95% confidence intervals (CIs).

**Results:**

The prevalence of CG in this population was 86%, while 66.7% of the study subjects screened positive for MDD. Significant predictors of MDD included age [OR 1.091, p=0.018], financial hardship [OR 9.47, p=0.014], accurate knowledge of the child’s prognosis [OR 0.268, p=0.046], perceived social support [(poor social support OR 6.402, P=0.039), (moderate social support OR 8.556, p=0.045)], and coping capacity [medium resilient copers OR 7.874, p=0.027]. Predictors of CG included age [OR 1.157, p=0.032], financial hardship [OR 11.501, p=0.04], years passed since child loss [1–2 years OR 4.634, p=0.049], and coping capacity [(low resilient copers OR 14.011, p<0.01), (medium resilient copers OR 19.023, p<0.01)].

**Conclusions:**

The study revealed high prevalence of MDD and CG among bereaved parents of pediatric cancer patients in Cameroon. Financial difficulty, social support, and coping capacity had substantial impact on parental mental health outcomes in this population. Personalized mental health support services into pediatric oncology care is critical for assisting bereaved families and encouraging resilience in the face of loss may improve health and wellbeing of the families. The study may have implications for global mental health in similar low-income countries.

## INTRODUCTION

Childhood cancers account for about 5% of all cancers in Africa, compared to the worldwide proportion of 1%^1^. In Cameroon, the incidence rate of childhood cancers stands at 25 cases per 1,000,000 children below 15 years annually. Children in this age group make up 42% of the Cameroon’s 27 million inhabitants, meaning each year, approximately 325 children in Cameroon are diagnosed with cancer^1^. Burkitt’s lymphomas are the most common childhood cancers in Cameroon with an incidence rate of 3.7 per 100,000 children annually. Other common childhood cancers in Cameroon include lymphomas, rhabdomyosarcomas, Wilms tumors, retinoblastomas, and Kaposi sarcomas^2^. Tragically, due to late diagnosis, limited treatment options, and financial constraints, nearly 80% of these children succumb to their illness within five years of diagnosis^2,3^. A child’s death is a devastating and unimaginable event, which usually leaves the surviving parents feeling helpless, guilty, shocked and in profound grief^3^. Bereavement grief is the term used to describe the constellation of debilitating and painful emotional, cognitive, behavioral, and somatic symptoms following the loss of a loved one^4^. It is a common, universal, physical, and psychological reaction to loss, and for most individuals typically decreases in intensity over time^5^. However, for a significant minority of individuals, the grieving process complicates into major depressive disorder (MDD), suicidal ideation^6^, or complicated grief (CG)^7^ also called prolonged grief disorder, defined as intense grief for more than 6 months.

The stress of caring for a severely ill loved one, coupled with their subsequent death predisposes bereaved parents to Major depressive disorder and Complicated Grief, and prevalence’s as high as 34% and 30% respectively have been reported in recent literature^6,7^. In Cameroon, as in most other countries in sub-Saharan Africa, parents often assume the primary caregiving role, staying with their children throughout hospital admissions, managing treatment regimens, and providing emotional support^1^. This caregiving responsibility, deeply rooted in cultural expectations, places significant psychological, social, and financial burdens on these parents, who often struggle with grief and mental health challenges long after their child’s passing^4,7^. Parents with CG suffer from increased risk of chronic physical and mental morbidities, with overall increased mortality rates^3^. One might as well say “they die from a broken heart”. Despite the relatively higher childhood cancer mortality rates in Cameroon, and the adverse health outcomes associated with parental grief, there is currently no study to the best of our knowledge aimed at measuring the prevalence of Major depressive disorder and complicated grief among this group of parents in Cameroon.

To gain some insight into this issue, we set out to determine prevalence of MDD and CG among bereaved parents of deceased pediatric cancer patients treated at Mbingo Baptist Hospital Cameroon, and to evaluate predictors of MDD and CG among the study participants.

## METHODS

### Study Design

This cross-sectional study included parents or primary caregivers of deceased pediatric cancer patients who had been treated at the Mbingo Baptist Hospital Cancer treatment center in Cameroon between January 2015 and January 2022. We obtained ethical clearance from the Cameron Baptist Convention Institutional Review Board. Eligible participants were identified by chart review, and the families were contacted by the hospital Chaplain who obtained verbal informed consent to the study.

### Study site

Mbingo Baptist Hospital is a 310-bed referral facility in the Northwest region of Cameroon, with 10 beds dedicated for in-patient treatment of pediatric cancer cases. Mbingo Baptist Hospital is one of the only two cancer treatment centers serving the Northwest region of the country.

### Subjects

We recruited parents or primary caregivers of deceased pediatric cancer patients who were at least 6 months post-loss, aged 18 or older, not on treatment for any mental health condition, and who gave their consent. We defined a childhood cancer patient as someone diagnosed with cancer by a physician in a health facility, and they were younger than 15 years at the time of diagnosis.

Primary caregivers included had been closely involved with the care and welfare of the child. For each child, only one parent or primary caregiver was recruited. Our sampling frame contained 105 eligible participants. To attain a 95% statistical power, we needed a minimum sample size of 83 participants. We accounted for a non-response rate of 10%, added 9 participants and set the new minimum sample size to 92 participants.

### Outcome variables

The outcome variables were major depressive disorder (MDD) and complicated grief (CG). We measured MDD using the Patient Health Questionnaire-9 (PHQ-9)^8^. This is a 9-item tool based on the 9 diagnostic criteria for MDD in the DSM-IV. Responses are scored on a Likert scale from 0 (not at all) to 3 (nearly every day). Final scores of 5, 10, 15, and 20 represent cutoffs for mild, moderate, moderately severe, and severe depression respectively.

We measured CG using the Brief Grief Questionnaire^9^. This is a standardized 5-item instrument used to screen for CG among bereaved individuals. The responses for each item are graded on a Likert scale from 0 (not at all) to 2 (a lot). A final score of 4 or more suggests that an individual may be suffering from CG and should prompt a referral to a grief specialist for further evaluation.

### Independent variables

We identified several variables from current literature which had been predictive of MDD and/or CG among study participants in settings like ours^10–15^.

Perceived social support; this was measured using the Oslo Social Support Scale (OSSS-3)^16^. This is a 3-item instrument that asks for number of close confidants, level of concern from other people and relationship with neighbors.

Resilience: this was measured using the brief resilience coping scale^17^, a 4-item instrument with 5-point scale responses and a total score ranging from 4–20. Participants who score between 4–13 are considered low resilient copers, those who score between 14–16 are considered medium resilient copers and those who score between 17–20 are high resilient copers.

Caregiver burden: this was measured using the short version of the Burden Scale for Family Caregivers (BCFC-s)^18^. This is a standardized highly reliable tool (Chronbach’s alpha = 0.92), with translations in 20 European languages indicating that it could be applied to a variety of settings. The questionnaire includes 10 questions with each question’s responses scored from 0–3. Scores were reported on a continuous scale with a possible range from 0–30.

Financial hardship: participants responded to the question ‘would you say you are experiencing financial hardship?’ with a ‘yes/maybe’ or a ‘no’.

Extent of suffering before death: participants described their perception of the extent of their child’s suffering before death as either minimal, moderate or extreme.

Accurate knowledge of child’s prognosis; this was measured by asking participants if they knew their child’s prognosis, and if so whether the taught the disease was curable, could reoccur, or that the child was probably going to die. Accurate knowledge of child’s prognosis was defined as participants choosing the latter option.

### Data collection

This was a quantitative study that involved the use of paper-based questionnaires. Ethical clearance was obtained from the Cameroon Baptist Convention’s Institutional Review Board on April 13, 2023. Chart review and participant recruitment began on May 10, 2023 and continued till July 12 2023. The questionnaire was tested for clarity and appropriateness in a pilot study involving 5 participants. Eligible participants were identified by reviewing charts of deceased children who had received treatment at the center between January 2015 and January 2022. The chaplain and palliative care nurse reached out to eligible participants by phone calls to present the study and obtained verbal consents for home visits. Verbally consented participants were administered the questionnaires in their homes by the chaplain and the palliative care nurse. Data collected was transferred into MS Excel, exported, and analyzed in SPSS version 28.

### Data analysis

We developed a multivariable binary logistic regression model in SPSS to identify Individual predictors. The dependent variables were Complicated Grief and Major Depressive Disorder, both of which were measured as binary variables. The model included all 7 predictor variables initially selected. The regression parameter for each predictor was reported as adjusted odds ratio (AOR) with a 95% level of confidence (CI = 95%).

## RESULTS

### Participant characteristic

92 participants completed the questionnaire. The mean age of the deceased at time of diagnosis was 10.7 years (S.D = 2.2). Proportion of unemployed participants was 56.5% (n=52), 65.6% (n=61) had some primary education and the overwhelming majority (90.1%, n=82) were Christians. Most participants had lost 2 children to all-causes (74.2%, n=66), and the responses to ‘number of living children’ were evenly distributed across 1–2 children (26.1%), 3–4 children (29.3%), and 5 or more children (34.8%) respectively. Most participants reported that the loss of their child to childhood cancer had occurred within the prior 2 years (Table 1).

### Prevalence of Major Depressive Disorder and Complicated Grief

**Fig 2.**
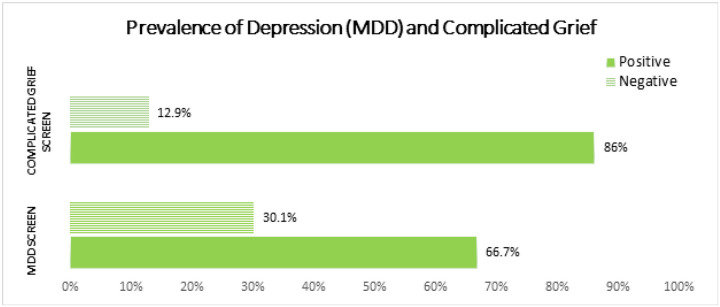
Prevalence of Major Depressive Disorder (MDD) and Complicated Grief.

**Figure 3:**
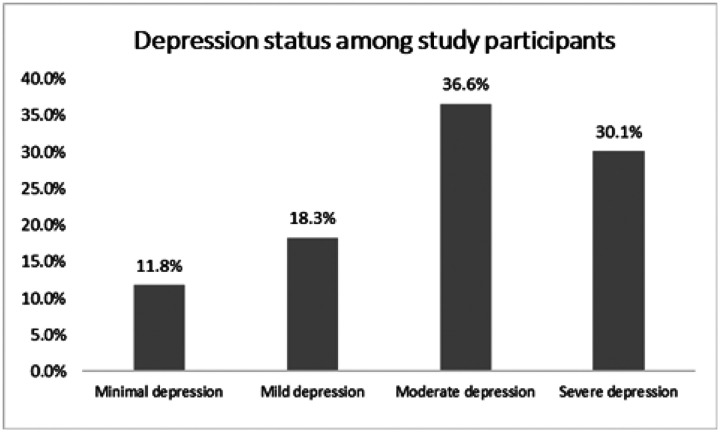
Depression types among study participants

Among study participants, 86% screened positive for CG (Figure 2). 11.8% were minimally depressed, and 18.3 were mildly depressed. 36.6% and 30.1% of participants screened positive for moderate and severe depression respectively (Figure 3). When the depression variable was dichotomized into MDD positive or not, 66.7% (n=62) of participants screened positive for MDD (Figure 2).

### Predictors for Major Depressive Disorder

Among study participants, age (aOR = 1.091, CI: 1.015–1.174, p=0.018), financial hardship (aOR 9.47, CI: 1.584 – 56.629, p=0.014), accurate knowledge of child’s prognosis at diagnosis (aOR 0.268, CI: 0.073 – 0.977), perceived social support and coping capacity were significant predictors of MDD. Participants who reported moderate and poor social supports were about 8 times (aOR 8.556 CI: 1.049–69.809) and 6 times (aOR 6.402 CI: 1.095 – 37.432) respectively more like to screen positive for MDD compared to those who reported strong social support. Medium-resilient and low-resilient copers were about 8 times (aOR 7.874 CI: 1.385 – 23.728, P = 0.027) and (aOR 8.401 CI: 0.854–82.686, P=0.068) respectively more likely to screen positive for MDD compared to high copers, although the latter was not statistically significant. Contrary to findings in current literature, years passed sine child loss, perception of extent of suffering before death and the burden of caregiving were not predictive of MDD among study participants (Table 2).

### Predictors for Complicated Grief

Age (aOR 1.157 CI: 1.084 – 1.230, p = 0.032), financial hardship (aOR 11.501, CI: 1.115 – 118.664, p = 0.046), years passed since child loss and coping capacity were significant predictors of screening positive for CG among study participants. Low resilient copers times (aOR 0.014 CI: 0.001 – 0.156, P<0.01) and medium resilient copers (aOR 0.019 CI: 0.002 – 0.019, p<0.01 were less likely to screen positive for CG compared to high resilient copers. Participants whose children had passed in the past 1–2 years were about 5 times (aOR 4.634 CI: 1.1012 – 210.191, P=0.049) more likely to screen positive for CG compared to those whose child had passed 5 or more years ago. Participants at 1-year and those at 3–4 years post loss did not have significantly higher risks for screening positive for CG (Table 2).

## DISCUSSION

Each year, at least 325 children in Cameroon are diagnosed with pediatric cancers, the most frequent of which are Burkitt lymphomas. Unfortunately, four out of every five of these youngsters will not live more than five years after their diagnosis. The traumatic impact of losing a child to cancer, along with the lack of proper support systems and resources, places enormous emotional and psychological loads on the surviving parents, predisposing them to MDD and CG. This is the first study to investigate the prevalence of MDD and CG among bereaved parents of pediatric cancer patients in Cameroon, as well as some common predictors of these outcomes.

### Prevalence of Major Depressive Disorder and Complicated Grief

Our study’s findings reveal a disturbingly high prevalence of MDD and CG respectively among bereaved parents. Approximately two-thirds of the grieving parents screened positive for MDD, while an overwhelming 86% had symptoms consistent with CG. These figures reaffirm the significant emotional suffering faced by parents after losing a child to cancer. Our study’s prevalence of MDD and CG substantially exceeds the national depression rate of 8.4% in Cameroon reported by Fodjo et al^19^ in 2021, underscoring the need for tailored interventions to support bereaved parents in Cameroon.

Other studies that examined depression and cg rates among bereaved family members and spouses found values ranging from 20–40% for CG^7,20–21^ and 26–34% for MDD^6,22–23^. Possible explanations for these significant differences include stronger attachments between parents and their children, the absence of any form of post-bereavement service among our participants, a practice that is very common in the West, where most reported studies were conducted, and our use of screening rather than diagnostic tools in our study. Despite the disparities in raw numbers, these studies convey the same story: mourning increases the probability of negative mental consequences after loss, emphasizing the need for focused therapies for bereaved parents.

### Predictors of Major Depressive Disorder and Complicated Grief

Several predictors emerged as significant contributors to the development of MDD and CG among the study participants. Financial hardship, accurate knowledge of the child’s prognosis at diagnosis, perceived social support, and coping capacity were among the key factors influencing parental mental health outcomes.

Financial stress appeared as a major predictor of both MDD and CG, emphasizing the link between socioeconomic issues and psychological well-being. Parents experiencing financial challenges may face increased stress and a sense of helplessness, increasing their susceptibility to mental health illnesses. Addressing financial barriers to getting healthcare and giving financial assistance to impacted families may ease some of the burdens that contribute to depressive symptoms and complicated grief disorder. These findings mirror those of Wen at al.^11^ in a 2022 study which showed that personal vulnerability (measured as financial hardship) among bereaved caregivers predisposed them to more distressing depressive symptom trajectories.

Accurate awareness of the child’s prognosis at the time of diagnosis was linked to a lower risk of MDD, highlighting the significance of open and honest communication between healthcare personnel and families. Parents who are well-informed about their child’s prognosis may feel more prepared and accepting, thus reducing psychological suffering caused by uncertainty and ambiguity around the outcome of their child’s condition. Our finding differed from those of Wen et al.^11^, who found no relationship between accurate knowledge of child’s prognosis and post-loss depression. More than eighty percent of our participants were religious. As a result, it is probable that they received signals of hope and recovery for their children during the treatment phase, which exacerbated their frustration when the child died.

Perceived social support emerged as a significant protective factor against MDD, with participants reporting strong social support being less likely to screen positive for depression. Previous literature has emphasized that social support helps in the grieving process^25^, and a lack of social support is a risk factor for negative bereavement outcomes^26^. According to Tompson et al.^27^, bereaved parents and siblings rely on family for social support from 6 to 19 months after losing a child to cancer. Pediatric oncology units play a vital role in assisting families during the palliative and grieving stages. A bereavement program should provide formal support services, communication from medical staff throughout palliation and after the child’s death, and opportunities to connect with other cancer-bereaved families for support^28^. Similarly, Nolbris and Hellström^29^ reported that friends are a vital source of social support for the bereaved.

Coping capacity, as evaluated by resilience, was also an important factor in predicting mental health outcomes. High resilient copers were less likely to test positive for CG than medium and low resilient copers. Building resilience through tailored interventions and psychotherapy approaches may help parents navigate the grief process more successfully and adaptively. These findings mirror those of Rasouli et al.^15^ in their 2022 study in which they found that higher resilience (measured by personal competence scores) was associated with lower rates of CG among bereaved siblings.

### Implications for practice and policy

The study’s findings have significant implications for clinical practice, policy creation, and resource allocation in pediatric cancer care in Cameroon and other similar countries. To begin, there is an urgent need to incorporate mental health support services into pediatric cancer care to meet the psychological needs of grieving parents. This could include educating healthcare providers about psychosocial care, developing grief support groups, and providing access to counseling and therapy services.

Furthermore, initiatives to improve communication and information exchange between healthcare practitioners and families can help to make better informed decisions and boost psychological readiness for end-of-life care. Investing in comprehensive palliative care services that stress holistic support for patients and their families, such as psychosocial and spiritual care, is critical to enhancing the quality of life for children with cancer and their caregivers.

On a policy level, there is a need to increase awareness of the psychosocial aspects of pediatric cancer care, as well as the necessity of mental health support for impacted families. Advocacy activities to raise mental health awareness, decrease stigma, and devote resources for psychosocial support services are crucial to meeting the unmet needs of bereaved parents and encouraging resilience in the face of loss.

### Study limitations

First, the participants were limited to parents who had lost a child at the Mbingo Baptist Hospital facility; therefore, the findings may not represent the entire population. Second, because of the nature of the study design, we cannot rule out recall bias. Third, the subjects who refused participation may have been suffering from severe grief or depression, although the response rate in the present study was 87.6%, which is a relatively high rate compared with previous studies^30^. Finally, assessing CG and MDD, we used the BGQ and the PHQ‐9, which are both screening tools and therefore may have over-estimated the true prevalence of MDD and CG among our study participants.

## CONCLUSION

Finally, this study sheds light on the tremendous emotional impact of children cancer on parents and caregivers in Cameroon. Identifying predictors of MDD and CG in bereaved parents gives useful insights for developing tailored therapies to help families cope with the death of a child to cancer. Addressing affected families’ psychosocial needs must be a top focus in pediatric oncology care, with coordinated efforts required to include mental health support services into existing healthcare systems and regulations.

## Figures and Tables

**Fig 1.**
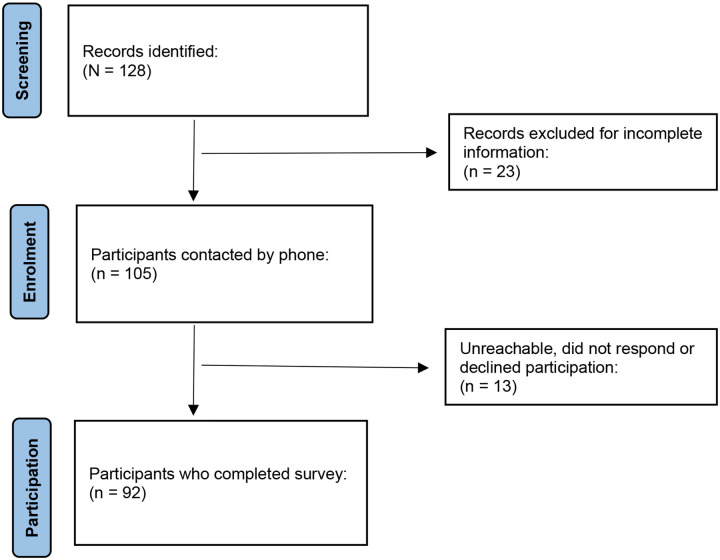
Flow chart of participant screening and enrollment

**Table 1:** Sociodemographic characteristics of participants

	n	%
**Participantstics of particip**	43	12
**Participantstics of participant**		
Full time	11	12.0
Part time	29	31.5
Unemployed/retired	52	56.5
**Participantretiredf participant**		
No formal education	15	16.1
Some primary education	61	65.6
Some secondary education	14	15.1
Some university education	2	3.2
**Financial hardship**		
Yes	74	81.3
No	17	18.7
**Participantardshipucat**		
Christian	82	90.1
Muslim	8	8.8
Traditional worship	2	1.1
Atheism	0	0
**Number of children lost**		
1 child	11	12.4
2 children	67	74.2
3 children	10	10.9
4 or more children	4	4.3
**Living children**		
None	9	9.8
1 – 2 children	24	26.1
3 – 4 children	27	29.3
5 or more children	32	34.8
**Participanthildren lostoncip**		
Single/divorced	28	30.8
Married	52	57.1
Widowed	12	12.1
**Deceased childdren l**		
Male	46	50
Female	46	50
**Childeed childdren lostoncipants**		
Less than 1 year	3	3.3
1 – 5 years	23	25.0
6 – 10 years	21	22.8
11 – 15 years	45	48.9
**Years passed since child loss**		
Less than 1 year	22	23.7
1 – 2 years	25	28.0
3 – 4 years	22	23.7
5 or more years	23	24.7

**Table 2:** Predictors of Major Depressive Disorder and Complicated Grief.

Independent variable	Major Depressive Disorder			Complicated grief		
	AOR	95% CI	p value	AOR	95% CI	p value
**Age**	1.091	1.015 – 1.174	**0.018**	1.157	1.012 – 1.322	**0.032**
**Financial hardship**						
Yes	9.47	1.584 – 56.629	**0.014**	11.501	1.115 – 118.664	**0.04**
No	Ref	Ref		Ref	Ref	
**Years passed since child loss**						
1 year	2.443	0.424 – 14.086	0.318	5.860	0.191 – 180.249	0.312
1 – 2 years	3.490	0.585 – 20.812	0.170	4.634	1.012 – 210.191	**0.049**
3 – 4 years	1.920	0.363 – 10.171	0.443	5.173	0.520 – 51.477	0.161
5 or more years	Ref	Ref		Ref	Ref	
**Accurate knowledge of childssDepressive Disorder and**						
Yes	0.268	0.073 – 0.977	**0.046**	0.195	0.016 – 2.441	0.205
No	Ref	Ref		Ref	Ref	
**Perception of extent of suffering before death**						
Minimal	0.039	0.001 – 1.217	0.065	2.285	0.001 – 1142.402	0.849
Moderate	0.514	0.085 – 3.099	0.468	0.761	0.037 – 15.523	0.859
Extreme	Ref	Ref		Ref	Ref	
Perceived social support						
Poor social support	6.402	1.095 – 37.432	**0.039**	1.896	0.199 – 17.584	0.585
Moderate social support	8.556	1.049 – 69.809	**0.045**	2.163	0.117 – 40.032	0.604
Strong social support	Ref	Ref		Ref	Ref	
**Coping capacity**						
Low resilient copers	8.401	0.854 – 82.686	0.068	14.011	1.136 – 156.867	**<0.01**
Medium resilient copers	7.874	1.385 – 23.728	**0.027**	19.023	2.537 – 109.001	**<0.01**
High resilient copers	Ref	Ref		Ref	Ref	
**Caregiver burden score**	1.049	0.983 – 1.119	0.149	1.105	0.981 – 1.245	0.1
